# Long-term survival with stable disease after multidisciplinary treatment for synchronous liver metastases from gastric cancer: A case report

**DOI:** 10.1016/j.ijscr.2019.08.021

**Published:** 2019-08-20

**Authors:** Wenjun Shi, Junfeng Wang, Wenjing Zhang, Tao Shou

**Affiliations:** a Department of Oncology, The First People’s Hospital of Yunnan Province, Kunming, China; b Department of General Surgical, The First People’s Hospital of Yunnan Province, Kunming, China

**Keywords:** CEA, carcino-embryonic antigen, CA 199, carbohydrate antigen 199, CA 724, carbohydrate antigen 724, CT, computed tomography, EBV, Epstein-Barr virus, ECOG PS, Eastern Cooperative Oncology Group performance status, FISH, fluorescence in situ hybridization, GCLM, gastric cancer with liver metastases, HER-2, human epidermal growth factor receptor-2, KPS, Karnofsky, L-ADM, liposome-entrapped Adriamycin, MDT, multidisciplinary team, MSI-H, microsatellite instability-high, NCCN, the National Comprehensive Cancer Network, PVC, portal vein chemotherapy, PEI, percutaneous ethanol injection, PD-L1, programmed death-ligand 1, PFS, progression free survival, TACE, transcatheter hepatic artery chemoembolization, RFA, radiofrequency ablation, RECIST, response evaluation criteria in solid tumors, S-1, tegafur, SD, stable disease, WHO, World Health Organization, Case report, Long-term survival, Gastric cancer, Liver metastases, Treatment

## Abstract

**Introduction:**

The liver is one of the most common sites of hematogenous metastases of gastric cancer. The 5-year overall survival rate of synchronous liver metastases from gastric cancer was less than 27%. We report a rare case of patient with synchronous liver metastases from gastric cancer who experienced stable disease for 7 years and 3 months following multidisciplinary modalities.

**The presentation of a case:**

A 33-year-old woman was admitted to our institute because of abdominal pain lasting for a day. Haemoglobin level was 68 g/L. Computed tomography (CT) scan revealed hemoperitoneum, multiple round lesions within liver parenchyma. The pathological diagnosis was gastric cancer with liver metastases. Following multidisciplinary treatment, she experienced stable disease for7 years and 3 months. Currently, the patient remains alive with no recurrence.

**Conclusion:**

We report a rare case of patient with synchronous liver metastases from gastric cancer who experienced stable disease for 7 years and 3 months following multidisciplinary modalities. Future trials are required to prospectively investigate the established regimen of multidisciplinary treatment.

## Highlights

• Based on the patients with better performance status, partial gastrectomy combining metastasectomy of liver is potentially useful in contributing to decrease tumor loading and cytokines secretion of tumor, which may result in immunosuppression.
• PVC has the potential to be a well-tolerated and excellent option in GCLM patients who have no extrahepatic metastases.
• Patients who without extrahepatic metastases, sensitive and well-tolerate to chemotherapy, having better performance status may get good prognosis.

## 1 Introduction

The liver, which via portal vein to transfer, is one of the most common sites of hematogenous metastases of gastric cancer. At the time of diagnosis, 35% of patients have distant metastases and 4–14% have metastases to the liver [[1], [2], [3]]. However, the treatment strategy for GCLM has not yet been established according to the NCCN Guidelines Version 2.2018. The prognosis for patients with liver metastases from gastric cancer is very poor and the 5-year overall survival rate of synchronous liver metastases from gastric cancer was less than 27% [[1], [2], [3]]. Some scholars reported GCLM patients with long-term survival, but no one live for more than 7 years [[4], [5], [6], [7], [8]].

Here, we report a patient with synchronous liver metastases from gastric cancer who experienced stable disease for more than 7 years following surgery, PVC, TACE, PEI, RFA, the administration of S-1. Currently, the patient remains alive with no recurrence. This case is reported with consideration to the SCARE criteria [9].

## 2 Presentation of the case

**Fig. 1:**
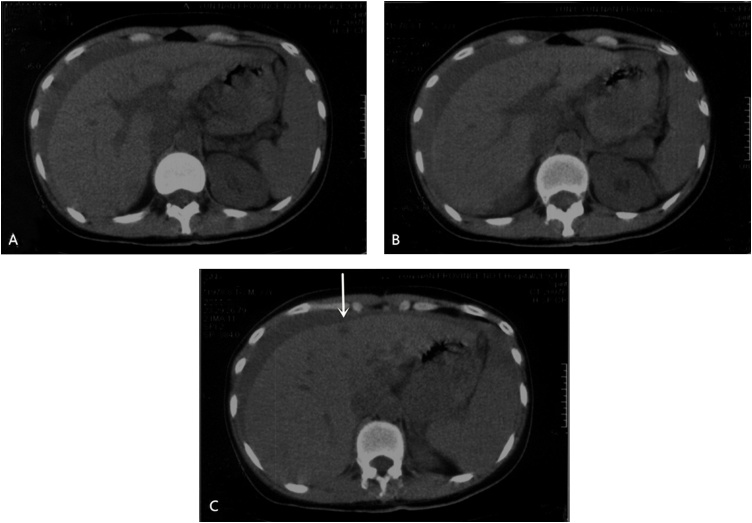
Immediate abdominal computed tomography revealing ascitic fluid in the peritoneal cavity, lymph node enlargement of hepatic portal (A, B) and a 19 × 13 mm^2^ metastatic lesion in the left medial segment of the liver (arrow) in August 2011.

A 33-year-old woman was admitted to our institute in August 2011 because of abdominal pain lasting for a day. She was well previously without any chronic comorbidity and did not have any family history of malignancy. On physical examination, she was haemodynamically stable with blood pressure at 110/70 mmHg and pulse rate 110/min. Her abdomen was soft with mild epigastric tenderness. Haemoglobin level was 68 g/L. Serum carcino-embryonic antigen (CEA), carbohydrate antigen 724 (CA 724) and carbohydrate antigen 199 (CA 199) levels were normal. An immediate abdominal CT scan revealed hemoperitoneum, multiple round lesions within liver parenchyma and lymph node enlargement of hepatic portal (Fig. 1).

**Fig. 2:**
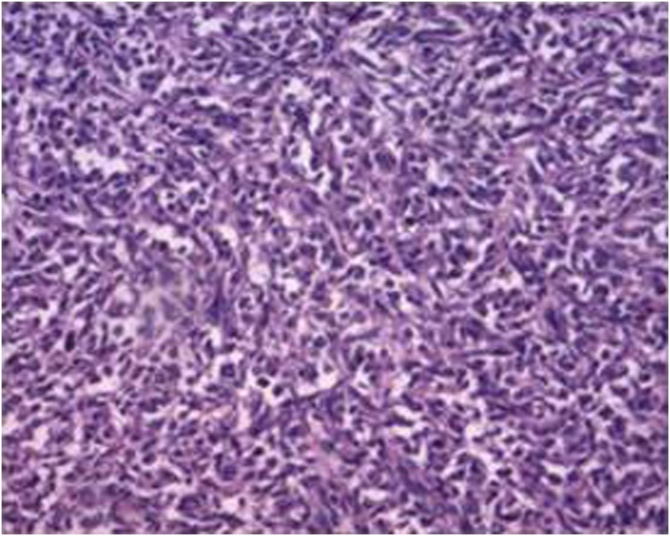
Histopathology of gastric cancer by biopsy showing undifferentiated carcinoma (hematoxylin and eosinstain; magnification, ×400).

The emergency exploratory laparotomy revealed linitis plastica, gastrorrhagia and liver lesions. Freezing tissue slices confirmed tumour cells. On the basis of these finding, gastric cancer with liver metastases was hypothesized based on clinical experience. The patient underwent partial gastrectomy, partial metastasectomy of left medial and lateral segment of liver. Microscopic examination confirmed undifferentiated gastric carcinoma (Fig. 2). The pathological diagnosis of the gastric cancer was T4aN1M1, Stage IV according to the NCCN Guidelines Version 1, 2011. Unfortunately, the patient refused to detect human epidermal growth factor receptor-2 (HER-2) gene expression examined by fluorescence in situ hybridization (FISH) due to financial burden.

**Fig. 3:**
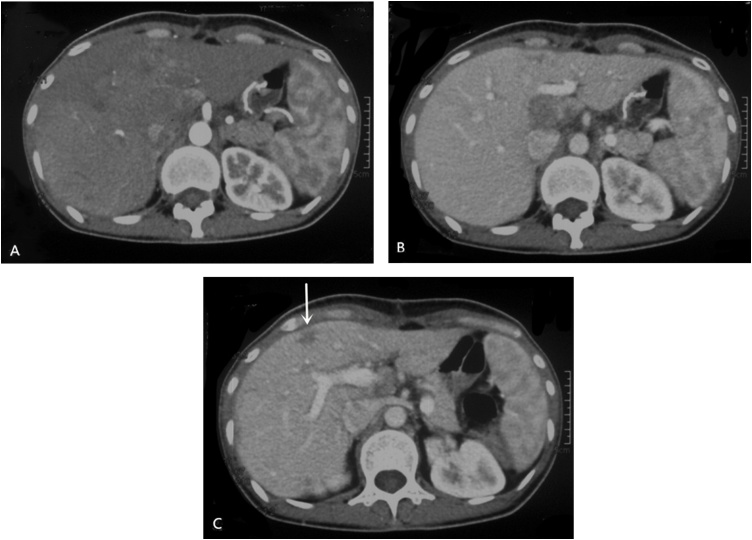
Abdominal computed tomography revealed that the tumor with stable disease after two courses of FOLFOX chemotherapy via the portal vein in October 2011.

Following the operation, the patient started 2 cycles of FOLFOX chemotherapy (oxaliplatin 85 mg/m^2^ IV on day 1, leucovorin 400 mg/m^2^ IV on day 1, fluorouracil 400 mg/m^2^ IV push on day 1, fluorouracil 1200 mg/m^2^ IV continuous infusion 46 h daily on day 1 and 2, cycled every 14 days) via the portal vein, and CT scan showed stable disease (SD) according to the response evaluation criteria in solid tumors (RECIST) 1.1 criteria (Fig. 3). Then, PVC was performed 43 months to prevent recurrence, the side effects was only mild marrow suppressed and gastrointestinal tract according to the toxic grading standard suggested by World Health Organization (WHO). Since the levels of the serum tumor marker (CEA, CA 724, CA 199) had small fluctuation (Fig. 5), the patient also received 8 courses of TACE, 2 courses of PEI, 1 course of RFA during this time. Sequentially, the administration of S-1 was continued up to December 2018.

## 3 Discussion

Although cooperative multidisciplinary team (MDT) may increase survival time and improve quality of life, the survival benefit is still unsatisfactory. The 5-year overall survival rate of synchronous liver metastases from gastric cancer was less than 27% [[1], [2], [3]]. Some scholars reported GCLM patients with long-term survival, but no one live for more than 7 years [[4], [5], [6], [7], [8]]. To the best of our knowledge, this is the first report of a patient with synchronous liver metastases from gastric cancer who experienced stable disease for more than 7 years following multidisciplinary treatment. Currently, the patient remains alive with no recurrence.

Liao [10] et al. reported that in their systematic review, gastrectomy combining metastasectomy of liver was associated with lower mortality rates in 677 patients of GCLM. Others also agreed on this point [[11], [12], [13]]. Similarly, our patient still alive with stable disease for more than 7 years. As this case suggests, based on the patients with better performance status [Karnofsky (KPS) score of ≥60% or Eastern Cooperative Oncology Group performance status (ECOG PS) score ≤2], partial gastrectomy combining metastasectomy of liver is potentially useful in contributing to decrease tumor loading and cytokines secretion of tumor, which may result in immunosuppression [14].

Since reporting the efficacy of systemic chemotherapies for gastric cancer, the number of patients undergoing PVC has been decreasing. Unlike systemic chemotherapy, PVC can not only destroy the remains of surrounding cancer cells, but also provide high concentrations of anticancer agents directly to the liver due to its slow velocity, that is, resulting in a decrease in adverse drug effects. Although no large-scale clinical trials evaluating the role of PVC in the treatment of advanced gastric cancer, PVC is one of the effective local treatments for liver metastases. It is also considered for patients who have no extrahepatic metastases and when the occurrence of hepatic metastases is expected to determine the patient’s prognosis. However, there is currently no established regimen of PVC. Mizuno [15] et al. reported that liposome-entrapped Adriamycin (L-ADM) (20–30 mg every 2 weeks) could be used for a long period (the average treatment period of PVC was 492 days) without severe adverse affects in 10 patients. Similarly, the long-term use of PVC with the long-term progression free survival (PFS) of our case described here, suggested that PVC have the potential to be a well-tolerated and excellent option in GCLM patients who have no extrahepatic metastases. Simultaneously, we also used TACE, PEI, RFA to interrupt blood supply. Sequentially, the administration of S-1, which can reduce adverse reactions of gastrointestinal and venous catheterization, was demonstrated to be a viable option in maintenance chemotherapy in advanced gastric cancer.

**Fig. 4:**
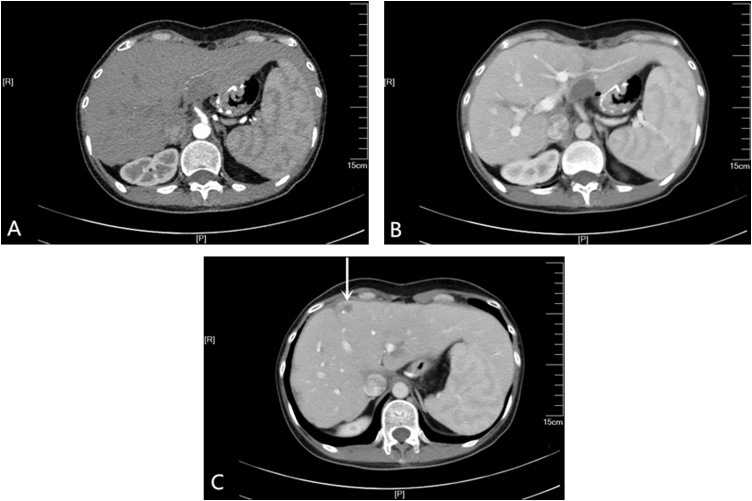
Abdominal computed tomography revealed that the tumor with stable disease in November 2018.

**Fig. 5:**
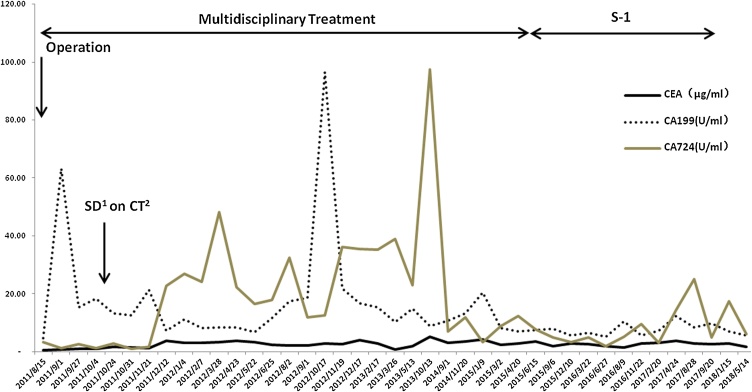
Clinical course and treatment of the patient. (1) Stable disease; (2) Computed tomography.

Because CT scan showed metastases with stable disease (Fig. 4) and the serum tumor marker (CEA, CA 724, CA 199) levels had no markedly elevated (Fig. 5) 7 years after multidisciplinary treatment, the patient still refuses to detect HER-2 gene expression examined, so we have not combined chemotherapy with targeted therapies. According to the NCCN Guidelines Version 2.2018, HER-2 gene and programmed death-ligand 1 (PD-L1) testing at the time of diagnosis are recommended for all advanced gastric adenocarcinoma patients if metastatic disease is documented or suspected. Several studies have shown that gastric cancers which are microsatellite instability-high (MSI-H) or Tumor Epstein-Barr virus (EBV)-positive show higher expression of PD-L1 compared to gastric cancers that do not show these traits [[16], [17], [18]]. Although, the prognostic significance of HER-2 status in patients with gastric cancer is unclear [19,20], systematic reviews and meta-analyses have shown that MSI-H and EBV-positive gastric cancers are associated with longer survival times and good prognosis [[21], [22], [23]].

As we all know, undifferentiated gastric carcinoma, particularly with synchronous liver metastases, has high malignancy and poor prognosis. Most patients who have gastric cancer and hepatic metastases are traditionally treated with palliative chemotherapy. The NCCN Guidelines Version 2.2018 recommend palliative gastrectomy and systemic chemotherapies for patients with advanced gastric carcinoma. Our individualized regimen, is different from others due to the better performance status (ECOG PS score ≤2) of this case, who had no extrahepatic metastases, especially sensitive and well-tolerate to chemotherapy.

## 4 Conclusion

Patients who without extrahepatic metastases, sensitive and well-tolerate to chemotherapy, having better performance status (KPS score of ≥60% or ECOG PS score ≤2) may get good prognosis. The patient described in this case report benefitted from the individualized regimen, as indicated by the long-term PFS and good safety. Thus, future trials are required to prospectively investigate the established regimen of multidisciplinary treatment.

## Sources of funding

This report did not receive any specific grant from funding agencies in the public, commercial, or not-for-profit sectors.

## Ethical approval

This article does not contain any personal information that can lead to the identification of the patient.

Our institution allows for exempt case reports as long as less than 5 participant in the case.

## Consent

Written informed consent was obtained from the patient for publication of this case report and accompanying images. A copy of the written consent is available for review by the Editor-in-Chief of this journal on request.

## Author’s contribution

Conceptualization: Wenjun Shi.

Data curation: Wenjun Shi.

Formal analysis: Wenjun Shi and Junfeng Wang.

Investigation: Wenjun Shi and Junfeng Wang.

Methodology: Wenjun Shi and Junfeng Wang.

Resources: Junfeng Wang.

Software: Wenjun Shi.

Supervision: Wenjing Zhang and Tao Shou.

Validation: Wenjing Zhang and Tao Shou.

Writing – original draft: Wenjun Shi and Junfeng Wang.

Writing – review and editing: Wenjun Shi and Junfeng Wang.

## Registration of research studies

N/A.

## Guarantor

Wenjun Shi and Junfeng Wang.

## Provenance and peer review

Not commissioned, externally peer-reviewed.

## Declaration of Competing Interest

All authors declare that they have no conflict of interest for this article.

## References

[1] Liu Q., Bi J.J., Tian Y.T., Feng Q., Zheng Z.X., Wang Z. (2015). Outcome after simultaneous resection of gastric primary tumour and synchronous liver metastases: survival analysis of a single-center experience in China. Asian Pac. J. Cancer Prev..

[2] Markar S.R., Mikhail S., Malietzis G., Athanasiou T., Mariette C., Sasako M. (2016). Influence of surgical resection of hepatic metastases from gastric adenocarcinoma on long-term survival: systematic review and pooled analysis. Ann. Surg..

[3] Song Ailin, Zhang Xiaofeng, Yu Feng, Li Debang, Shao Wenyu, Zhou Yanming (2017). Surgical resection for hepatic metastases from gastric cancer: a multi-institution study. Oncotarget.

[4] Kiyota S., Matsuda Y., Hanada S., Kinoshita M., Nishizawa S., Kanazawa G. (2016). A case of long-term survival after combination therapy for gastric cancer with synchronous multiple liver metastases. Gan To Kagaku Ryoho.

[5] Saitoh H., Boku N., Ohtsu A., Hironaka S., Miyamoto S., Hamamoto Y. (2000). Five-year survivor with liver metastases from gastric cancer successfully treated with systemic chemotherapy. Gastric Cancer.

[6] Masuzawa T., Fujiwara Y., Takiguchi S., Yamazaki M., Miyata H., Nakajima K. (2008). A long-term survival case of gastric cancer with liver metastases treated by hepatic arterial infusion chemotherapy. Gan To Kagaku Ryoho.

[7] Koyasaki N., Matsumura A., Kamata T., Kanno M. (2002). A case of advanced gastric carcinoma with liver metastases with no recurrence and long survival by means of surgery and postoperative chemotherapy. Gan To Kagaku Ryoho.

[8] Barchiellia A., Amorosib A., Balzia D., Crocettic E., Nesib G. (2001). Long-term prognosis of gastric cancer in a European country: a population-based study in Florence (Italy). 10-Year survival of cases diagnosed in 1985–1987. Eur. J. Cancer.

[9] Agha R.A., Borrelli M.R., Farwana R., Koshy K., Fowler A., Orgill D.P., For the SCARE Group (2018). The SCARE 2018 statement: updating consensus surgical case report (SCARE) guidelines. Int. J. Surg..

[10] Liao Y.Y., Peng N.F., Long D., Yu P.C., Zhang S., Zhong J.H. (2017). Hepatectomy for liver metastases from gastric cancer: a systematic review. BMC Surg..

[11] Markar S.R., Mackenzie H., Mikhail S., Mughal M., Preston S.R., Maynard N.D. (2017). Surgical resection of hepatic metastases from gastric cancer: outcomes from national series in England. Gastric Cancer.

[12] Long D., Yu P.C., Huang W., Luo Y.L., Zhang S. (2016). Systematic review of partial hepatic resection to treat hepatic metastases in patients with gastric cancer. Medicine.

[13] Oki E., Tokunaga S., Emi Y., Kusumoto T., Yamamoto M., Fukuzawa K. (2016). Surgical treatment of liver metastases of gastric cancer: a retrospective multicenter cohort study (KSCC1302). Gastric Cancer.

[14] Pollock E.R., Roth A.J. (1989). Cancer-induced irnmunosuppression: implications for therapy?. Semin. Surg. Oncol..

[15] Mizuno I., Ichino T., Yotsuyanagi T., Akamo Y., Yamamoto T., Yasui T. (1991). Clinical application of chemotherapy via the portal vein with liposome-encapsulated adriamycin in inoperable metastatic liver cancer. Gan To Kagaku Ryoho.

[16] Ma C., Patel K., Singhi A.D., Ren B., Zhu B., Shaikh F. (2016). Programmed death-ligand 1 expression is common in gastric cancer associated with Epstein-Barr virus or microsatellite instability. Am. J. Surg. Pathol..

[17] Derks S., Liao X., Chiaravalli A.M., Xu X., Camargo M.C., Solcia E. (2016). Abundant PD-L1 expression in Epstein-Barr virus-infected gastric cancers. Oncotarget.

[18] Cho J., Lee J., Bang H., Kim S.T., Park S.H., An J.Y. (2017). Programmed cell death-ligand 1 expression predicts survival in patients with gastric carcinoma with microsatellite instability. Oncotarget.

[19] Chua T.C., Merrett N.D. (2012). Clinicopathologic factors associated with HER2-positive gastric cancer and its impact on survival outcomes—a systematic review. Int. J. Cancer.

[20] Kunz P.L., Mojtahed A., Fisher G.A., Ford J.M., Chang D.T., Balise R.R. (2012). HER2 expression in gastric and gastroesophageal junction adenocarcinoma in a US population: clinicopathologic analysis with proposed approach to HER2 assessment. Appl. Immunohistochem. Mol. Morphol..

[21] Zhu L., Li Z., Wang Y., Zhang C., Liu Y., Qu X. (2015). Microsatellite instability and survival in gastric cancer: a systematic review and meta-analysis. Mol. Clin. Oncol..

[22] Choi Y.Y., Bae J.M., An J.Y., Kwon I.G., Cho I., Shin H.B. (2014). Is microsatellite instability a prognostic marker in gastric cancer? A systematic review with meta-analysis. J. Surg. Oncol..

[23] Cho J., Kang M.S., Kim K.M. (2016). Epstein-Barr virus-associated gastric carcinoma and specific features of the accompanying immune response. J. Gastric Cancer.

